# Lama2 And Samsn1 Mediate the Effects of Brn4 on Hippocampal Neural Stem Cell Proliferation and Differentiation

**DOI:** 10.1155/2023/7284986

**Published:** 2023-04-13

**Authors:** Lei Zhang, Xunrui Zhang, Ruijie Ji, Yaya Ji, Yuhang Wu, Xiuyu Ding, Zhiying Shang, Xueyuan Liu, Wen Li, Jingjing Guo, Jue Wang, Xiang Cheng, Jianbing Qin, Meiling Tian, Guohua Jin, Xinhua Zhang

**Affiliations:** ^1^Department of Anatomy, Institute of Neurobiology, Medical School, Co-Innovation Center of Neuroregeneration, Nantong University, Nantong, China; ^2^Faculty of Medicine, Xinglin College, Nantong University, Nantong, China; ^3^Central Lab, Yancheng Third People's Hospital, The Sixth Affiliated Hospital of Nantong University, Yancheng 224002, China

## Abstract

The transcription factor Brn4 exhibits vital roles in the embryonic development of the neural tube, inner ear, pancreas islet, and neural stem cell differentiation. Our previous studies have shown that Brn4 promotes neuronal differentiation of hippocampal neural stem cells (NSCs). However, its mechanism is still unclear. Here, starting from the overlapping genes between RNA-seq and ChIP-seq results, we explored the downstream target genes that mediate Brn4-induced hippocampal neurogenesis. There were 16 genes at the intersection of RNA-seq and ChIP-seq, among which the Lama2 and Samsn1 levels can be upregulated by Brn4, and the combination between their promoters and Brn4 was further determined using ChIP and dual luciferase reporter gene assays. EdU incorporation, cell cycle analysis, and CCK-8 assay indicated that Lama2 and Samsn1 mediated the inhibitory effect of Brn4 on the proliferation of hippocampal NSCs. Immunofluorescence staining, RT-qPCR, and Western blot suggested that Lama2 and Samsn1 mediated the promoting effect of Brn4 on the differentiation of hippocampal NSCs into neurons. In conclusion, our study demonstrates that Brn4 binds to the promoters of Lama2 and Samsn1, and they partially mediate the regulation of Brn4 on the proliferation inhibition and neuronal differentiation promotion of hippocampal NSCs.

## 1. Introduction

Neural stem cells (NSCs) exist in the mammalian brain throughout their life, mainly distributed in the subgranular zone (SGZ) of the hippocampal dentate gyrus and subventricular zone (SVZ) of the lateral ventricle [1]. After migration and differentiation, NSCs in SGZ eventually mature into granular cells in the dentate gyrus. On the one hand, they receive neural afferents from the entorhinal cortex. On the other hand, they can also form axons to project to pyramidal cells in the hippocampal CA3 region. This is an important part of the hippocampal “trisynaptic circuit”, which is closely related to learning and memory processes [1, 2]. Therefore, the self-renewal and differentiation of NSCs in the SGZ directly affect the maintenance and repair of hippocampal function.

Alzheimer's disease (AD) is an age-related chronic degenerative disease of the central nervous system. The main pathological features in AD brain tissues are the extracellular diffuse or plaque-like abnormal deposition of *β*-amyloid protein and the intracellular neurofibrillary tangles caused by the hyperphosphorylation of tau protein in neurons [3]. In addition, the progression of AD is also closely related to astrocytes, microglia, vascular system, mitochondrial dysfunction, inflammation, intestinal flora, and immune system [4–6]. These factors work together to cause synaptic dysfunction and neuronal degeneration in the brain of AD patients, which trigger a series of neuropsychiatric symptoms and eventually death [7]. However, till now, there is no effective treatment for the loss of neurons and the consequent neurological symptoms in AD patients [3]. During the progression of AD, the proliferation, migration, and differentiation of hippocampal NSCs are significantly changed, suggesting that hippocampal NSCs may be closely related to the pathogenesis of AD, like synaptic abnormalities, amyloid deposition, neuronal loss, and the changes in learning and memory ability [8–10]. Although neurogenesis seems to increase in AD patients, progressive neuron loss is still observed, which indicates that the differentiation ability of NSCs in AD brain is insufficient [8].

Since the transplantation of human fetal substantia nigra dopaminergic neurons into a rat model of Parkinson's disease significantly improved the disease behavior [11], cell replacement therapy has gradually become a research hotspot as a breakthrough to solve major nervous system diseases. Transplanted cells come from a wide range of sources, in which NSCs are the most direct source of neurons and glial cells. Other pluripotent stem cells, such as embryonic stem cells and mesenchymal stem cells, first need to be transformed into neural lineage precursor cells to differentiate into neurons, while NSCs can bypass this step and directly differentiate into required neurons or glial cells. In addition, compared with other pluripotent stem cells, NSCs also largely avoid the risk of teratogenesis after transplantation [12]. However, the proportion of NSCs differentiating into neurons is low, which cannot meet the number requirements of cell transplantation under pathological conditions [13]. Therefore, clarifying the regulation mechanism of NSC proliferation and differentiation may provide new ideas and methods not only for the brain self-repairing but also for cell replacement therapy.

The transcription factor Brn4, namely, Brain 4, also known as POU3F4, OCT9, OTF9, DFN3, and DFNX2, is named because of its first discovery in the brain and plays an important role in embryonic development, cell reprogramming, and regulation of NSC proliferation and differentiation [14]. Brn4 can promote not only the differentiation of NSCs into neurons but also the maturation of newborn neurons [15–18]. Besides, many studies have shown that Brn4 can also reprogram human or mouse fibroblasts and astrocytes into neurons or neuron precursor cells [19–22]. All these researches suggest that the role of Brn4 in the development of the central nervous system and the differentiation of NSCs should not be ignored.

The behavior experiments also showed that the learning and memory abilities of Brn4 knockout mice decreased significantly. At the same time, Brn4 not only inhibited the proliferation of hippocampal NSCs but also promoted their differentiation into neurons. These results suggest that Brn4 may have a protective effect on the brain during the progression of AD. However, the mechanism of Brn4 regulating the proliferation and differentiation of hippocampal NSCs is still unclear. RNA sequencing analysis suggested that the level of genes related to neuron development and maturation increased, while the level of genes related to the maintenance of NSC stemness decreased after Brn4 overexpression [23], indicating the existence of downstream genes mediating the function of transcription factor Brn4. In this study, we verified the intersection genes of RNA-seq and ChIP-seq results through multiple experiments to explore the downstream target genes directly bound by Brn4 and the molecular mechanism of Brn4 regulating the neurogenesis in the hippocampus. Our research reveals the downstream mechanism of the transcription factor Brn4, and it may provide new ideas for regulating the proliferation and differentiation of NSCs *in vitro* and *in vivo*, which is helpful to solve neurodegenerative diseases through self-repairing or cell replacement therapy.

## 2. Materials and Methods

### 2.1. Animals and Cell Culture

The SPF neonatal (1 day) ICR mice used for the primary culture of hippocampal NSCs were purchased from the Animal Research Center of Nantong University of China. We made great efforts to reduce the number of mice sacrificed and to minimize their pain. All animal experiments were approved by the Nursing and Use Committee of the Experimental Animal Research Center of Nantong University. The N2a cell line was obtained from the National Collection of Authenticated Cell Cultures. N2a cells were cultured in Dulbecco Modified Eagle Medium and Ham F12 (DMEM/F12, Corning) supplemented with 10% FBS (Gibco) and 100 U/mL penicillin-streptomycin (HyClone) at 37°C with 5% CO_2_. The culture and differentiation of hippocampal NSCs were performed as described previously with some modifications [24, 25]. Firstly, the hippocampi were derived from the brains of neonatal (1 day) ICR mice and mechanically dissociated in DMEM/F12. After the cell suspension passed through a 200-mesh nylon filter, single cells were cultured in a 25 cm^2^ flask at a density of 1 × 10^4^ cells/mL with NSCs culture medium, which was composed of DMEM/F12, 2% B27 (Gibco), 20 ng/mL epidermal growth factor (EGF, Gibco), 20 ng/mL fibroblast growth factor 2 (bFGF, Sigma) and 100 U/mL penicillin/streptomycin (Shanghai Biotech Research Institute) at 37°C in a humidified incubator with 95% air and 5% (v/v) CO_2_. Secondly, about 5-7 days later, neurospheres were formed and suspended in the medium. Using the Accutase enzyme (Sigma), we digested these spheres into single cells and continued to culture. Thirdly, after three generations, single cells at a density of 1 × 10^4^ cells/mL were plated in 24-well plates coated with poly-L-lysine (Sigma) in advance. Some were cultured with an NSC culture medium, and others were cultured with a differentiation medium composed of DMEM/F12 and 1% fetal bovine serum (FBS, Gibco). Finally, 7 days later, in order to determine whether the cells we cultured were NSCs, immunocytochemistry was used to detect the expression of stem cell markers and various neural cell markers, respectively, to detect the characterization of the cells. So as to ensure the purity of cells, we used neurospheres of the third generation to carry out subsequent experiments.

### 2.2. Plasmid Transfection and Virus Infection

Lama2 CRISPR SAM coactivated plasmids (Santa Cruz) were used to activate the Lama2 expression. After being seeded onto 6-well plates with 3 mL medium for each well, cells were transfected, respectively, with 1 ug, 2 ug, or 3 ug plasmids by Lipofectamine 3000 (Invitrogen) according to the manufacturer's protocol. The lentiviruses were directly added into the medium to infect cells. The overexpression and knockdown lentiviruses used in this study were as follows: pLenti-EF1a-EGFP-F2A-Puro-CMV-MCS (negative control) vs. pLenti-EF1a-EGFP-F2A-Puro-CMV-Brn4-HA (Genechem); pSLenti-EF1a-EGFP-F2A-Puro-CMV-MCS-3Flag (negative control) vs. pSLenti-EF1a-EGFP-F2A-Puro-CMV-Samsn1-3Flag (OBiO); pSLenti-U6-shRNA-CMV-EGFP-2A-Puro (negative control) vs. pSLenti-U6-shRNA(Lama2)-CMV-EGFP-2A-Puro (Lama2-shRNA) (OBiO); pSLenti-U6-shRNA-CMV-EGFP-2A-Puro (negative control) vs. pSLenti-U6-shRNA (Samsn1)-CMV-EGFP-2A-Puro (Samsn1-shRNA) (OBiO); and pLKD-CMV-mcherry-2A-Puro-U6-shRNA (negative control) vs. pLKD-CMV-mcherry-2A-Puro-U6-shRNA(Pou3f4) (Brn4-shRNA) (OBiO).

### 2.3. Establishment of Stable Lentivirus Strains

2 × 10^5^ N2a cells were plated onto a 6-well plate and cultured at 37°C with 5% CO_2_ for 12-16 h. When the cell confluence reached approximately 40-60%, the control lentivirus (empty vector, pLenti-EF1a-EGFP-F2A-Puro-CMV-MCS) and Brn4 overexpression lentivirus (pLenti-EF1a-EGFP-F2A-Puro-CMV-Brn4-HA) were, respectively, added into the medium and cultured for 48 h. Then, the cells were selected with 2 *μ*g/mL puromycin (YEASEN) for 7 days. RT-qPCR and Western blot were performed to detect the Brn4 overexpression efficiency.

### 2.4. ChIP Assay

4 × 10^6^ Brn4-overexpressing N2a cells were needed for each immunoprecipitation. The ChIP assay was carried out according to the manufacturer's instructions (Cell Signaling Technology). Briefly, the cells were cross-linked by 1% formaldehyde, and then the chromatin was digested to 150-900 bp DNA-protein fragments by micrococcal nuclease and ultrasonic treatment. After respective incubation with different immunoprecipitating antibodies overnight at 4°C, elution from antibody/protein G agarose beads, and reversal of cross-links, the DNA was purified using spin columns and quantified by PCR. PCR was performed with DreamTaq Green PCR Master Mix (Thermo Fisher Scientific) according to the manufacturer's protocol using the following PCR conditions: 95°C for 5 min, followed by 40 cycles of 95°C for 15 s, 55°C for 30 s, and 72°C for 40 s. The results were visualized using 1% agarose gel electrophoresis. The sequences of the primers used in the study are in Table 1.

### 2.5. Dual Luciferase Reporter Gene Experiment

The binding sites of Brn4 on the Lama2 and Samsn1 promoters were analyzed using the JASPAR website (http://jaspar.genereg.net/). The top three sites with the highest scores were deleted to construct the mutated dual luciferase reporter gene plasmid, respectively. 1 × 10^4^ N2a cells were plated onto 96-well plates and cultured overnight at 37°C with 5% CO_2_. 48 h after the plasmids (OBiO) transfection by Lipofectamine 3000 (Invitrogen), the dual luciferase reporter gene experiment was performed by using the Duo-Lite Luciferase Assay System (Vazyme) according to the manufacturer's protocol. The luminescence was measured using Synergy 2 multimode reader (Gene Company).

### 2.6. RNA Extraction and RT-qPCR

For the stable N2a lentivirus strains, RNA extraction was performed until the cell confluence reached approximately 80%. For primary hippocampal NSCs, the RNA was extracted 7 days after induced differentiation in DMEM/F-12 supplementing with 1% FBS and 100 U/mL penicillin-streptomycin 24 h after plasmid transfection or 48 h after lentivirus infection at 37°C with 5% CO_2_. As described previously [26], total RNA was extracted using Total RNA Isolation Reagent (Shanghai Pufei Biotechnology), cDNA synthesis was performed with a RevertAid First Strand cDNA Synthesis kit (Thermo Fisher Scientific), and RT-qPCR was performed with Universal SYBR Green Master Mix (Roche Diagnostics) according to the manufacturer's protocol using the following PCR conditions: 95°C for 10 min, followed by 40 cycles of 95°C for 30 s, 60°C for 30 s and 72°C for 40 s, followed by a final elongation step of 10 min at 72°C. Gene expression was normalized to *β*-actin expression calculated using the 2^−ΔΔCT^ method [27]. The primer sequences are shown in Table 2.

### 2.7. Western Blot

The cells were harvested when N2a stable lentivirus strains reached 80-90% confluence or primary hippocampal NSCs were induced to differentiate for 7 days using DMEM/F-12 supplemented with 1% FBS 24 h after plasmid transfection or 48 h after lentivirus infection at 37°C with 5% CO_2_. In brief, cell monolayers were washed with PBS and harvested in Mammalian Protein Extraction Reagent (Thermo Fisher Scientific) containing Protease Inhibitor Cocktail (Thermo Fisher Scientific). Equal amounts of protein were fractionated in SDS-PAGE gels (Epizyme Biotech) and transferred onto a 0.45 *μ*m PVDF membrane. The membranes were probed at 4°C overnight after being blocked in 5% skim milk-TBST with the following primary antibodies: anti-*β*-actin (Abcam), anti-Brn4 (Abcam), anti-Lama2 (Abcam), and anti-Tuj1 (Abcam) at 1 : 1,000; anti-GFAP (Abcam) at 1 : 2,000; anti-Samsn1 (Proteintech) at 1 : 3,000; anti-HA (Sigma) at 1 : 20,000. Then, incubation was performed for 2 h at room temperature (RT) with respective HRP-labeled secondary antibodies (Abcam) diluted at 1 : 5,000. The results were visualized by ChemiDoc Touch Imaging System (Bio-Rad) with Clarity Western ECL Substrate (Bio-Rad). The protein expression was normalized to *β*-actin bands.

### 2.8. EdU Incorporation

A Cell-Light EdU Apollo567 in vitro kit (RiboBio) was used for EdU combination assay according to the manufacturer's protocol. The cells were incubated in the EdU medium for 2 h at 37°C 24 h after plasmid transfection or 48 h after lentivirus infection. After double staining with Hoechst (Beyotime) cells were analyzed under a fluorescence microscope (Zeiss AG) using x400 magnification. EdU and Hoechst-labeled cells in the 10 random fields were counted, and the percentage of EdU/Hoechst was calculated for comparison between groups.

### 2.9. Cell Cycle Analysis

As described [26], NSCs were harvested 24 h after plasmid transfection or 48 h after lentivirus infection and then fixed with ice-cold 75% ethanol at -20°C for at least 24 h. Next, cells were stained with Hoechst 33342 solution (BD Biosciences) and incubated for 30 min at RT, washed, and suspended in BD Pharmingen™ Stain Buffer (BD Biosciences). The results were analyzed using the BD FACSCalibur system (BD Biosciences) with ModFit LT v3.3.11 software (Verity Software House).

### 2.10. CCK-8 Assay

Cell Counting Kit-8 (Dalian Meilun Biology Technology) was used in the CCK-8 assay following the manufacturer's instructions. 1 × 10^4^ NSCs were plated in poly-L-lysine-coated (Sigma) 24-well plates and cultured at 37°C for 24 h. Then, the cells were infected or transfected, respectively, with the lentiviruses or plasmids. After the infection or transfection for 0, 1, 2, 3, and 4 days, 50 *μ*L CCK-8 reagent was added to each well and incubated at 37°C for 2 h. The optical density (OD) was examined at a wavelength of 450 nm (OD 450 nm) using the Synergy 2 multmode reader.

### 2.11. Immunofluorescence Staining

The primary hippocampal NSCs were induced to differentiate for 7 days using DMEM/F-12 supplemented with 1% FBS 24 h after plasmid transfection or 48 h after lentivirus infection at 37°C with 5% CO_2_. Briefly, cell monolayers were washed with PBS and fixed in 4% formaldehyde for 20 min at RT. After being blocked in 10% goat serum-PBS containing 0.3% Triton X-100, the cells were in turn incubated at 4°C overnight with anti-Tuj1 antibody (Abcam), at RT for 2 h with Alexa Fluor 555-labeled secondary antibodies (Invitrogen), and at RT for 10 min with Hoechst (Beyotime). The primary antibodies were as follows: mouse anti-microtubule associated protein (MAP) 2 (1 : 200), mouse anti-Nestin (1 : 100) (both from Millipore); rabbit anti-glial fibrillary acidic protein (GFAP, 1 : 1,000), rabbit anti-*β*-III-tubulin (Tuj1, 1 : 1,000) (both from Abcam); rabbit anti-Ki67 (1 : 200, Sigma). The secondary antibodies were Alexa Fluor 568-conjugated goat anti-rabbit IgG (1 : 1,000) and Alexa Fluor 488-conjugated goat anti-mouse IgG (1 : 1,000) (both from Invitrogen).

### 2.12. Statistical Analysis

GraphPad Prism 8.0 software (GraphPad Software, La Jolla, CA, USA) was used for statistical analysis. The data were presented as the mean ± SEM based on at least three independent samples in each group. Experiments with two experimental groups were evaluated using an unpaired Student's two-tailed *t*-test. In experiments with more than two experimental groups, one-way analysis of variance was used. *P* < 0.05 was considered statistically significant.

## 3. Results

### 3.1. Culture and Identification of Hippocampal NSCs

**Figure 1:**
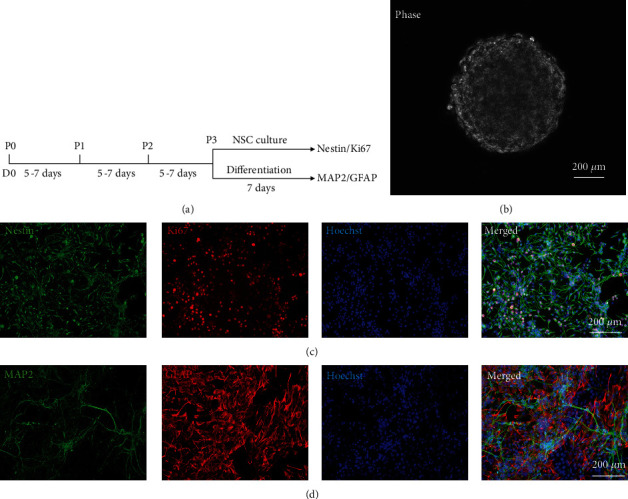
Culture and identification of hippocampal NSCs. (a) The schematic of the culture and differentiation of NSCs. (b) A representative image showing the morphology of the third-generation neurosphere derived from the hippocampi of the neonatal (1 day) ICR mice. (c) Immunocytochemistry staining on the third generation showed that most of the cells expressed Nestin (green). and Ki67 (red). (d) After being cultured in a differentiation medium for 7 d, immunocytochemistry analysis showed that some cells could express MAP2 (green) and GFAP (red). Nuclei were stained with Hoechst (blue). Scale bar = 200 *μ*m.

The process of cell culture and differentiation was conducted as shown in the schematic in Figure 1(a). The single cells digested from the third generation of neurospheres (Figure 1(b)) were cultured in an NSC culture medium for 7 days. Immunocytochemistry analysis showed that about 95.47% of cells were Nestin-positive (Figure 1(c)), indicating that these cells were embryonic. And about 98.35% of cells were positive for Ki67 (Figure 1(c)), indicating that cells were in the state of proliferation. To further determine the multiple differentiation potential, the single cells of the third generation were cultured in the differentiation medium for 7 days. Immunocytochemistry analysis showed that some cells could express MAP2 or GFAP (Figure 1(d)), which indicated that cells had the ability to differentiate into neurons and astrocytes. Therefore, we considered that the cells we extracted from the hippocampus and purified were NSCs.

### 3.2. Brn4 Bound to the Promoters of Lama2 and Samsn1 and Promoted Their Expression

**Figure 2:**
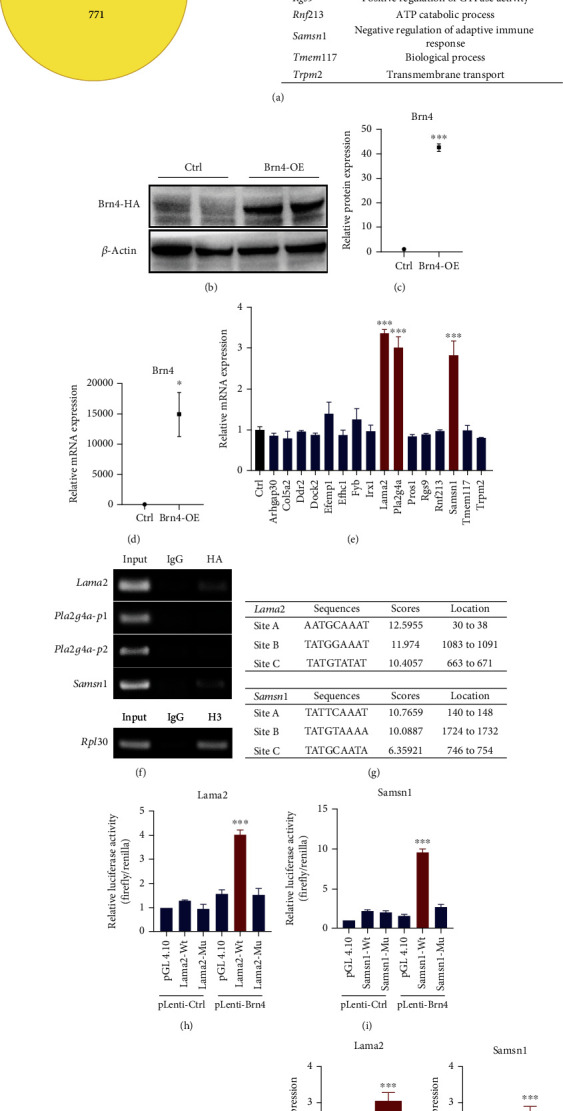
Brn4 binds to the promoters of Lama2 and Samsn1 and promotes their expression. (a) Overlapping genes of RNA-seq and ChIP-seq. (b, c) Representative immunoblots of Brn4-HA and *β*-actin in the N2a stable Brn4-overexpressed lentivirus strains (b) and quantification of Brn4-HA protein level normalized to *β*-actin (c). (d) Quantification of Brn4 mRNA level normalized to *β*-actin. (e) Quantification of mRNA levels for the overlapping genes between RNA-seq and ChIP-seq normalized to *β*-actin. (f) Nucleic acid electrophoresis for ChIP-PCR assay in the N2a stable Brn4-overexpressed lentivirus strains. (g) Sequences of the top three Brn4 binding sites on the Lama2 and Samsn1 promoters with the highest scores. (h, i) Dual-luciferase reporter gene experiment using N2a cells. (j–m) Representative immunoblots of Lama2, Samsn1, and *β*-actin in the N2a stable Brn4-overexpressed lentivirus strains (j, k) and quantification of Lama2 and Samsn1 protein levels normalized to *β*-actin (l, m). Error bars represent the mean ± SEM. *n* = 3. ^∗^*P* < 0.05, ^∗∗∗^*P* < 0.001. Abbreviations: Ctrl: control; Brn4-OE: Brn4-overexpression.

Previous studies have shown that transcription factor Brn4 can promote the differentiation of hippocampal NSCs into neurons [15, 16], but the specific mechanism is not clear. Therefore, we used RNA-seq and ChIP-seq to detect the changes in the gene expression level of the cells after forced Brn4 overexpression. The RNA-seq data were analyzed with FDR < 0.001 and Fold Change ≥ 2 as screening criteria, and the results showed that the transcription level of 638 genes elevated after Brn4 overexpression. The ChIP-seq data were analyzed with *P* value < 0.001 as the screening criteria, and the results showed that 771 genes were involved in the Brn4 binding sequence. There were totally 16 genes in the intersection of the two sequencing results (Figure 2(a)). In this study, we took the 16 genes, overlapping genes of RNA-seq and ChIP-seq, as the breakthrough to explore the exact downstream target genes bound by Brn4.

At first, we established two different stable lentivirus strains (Brn4 overexpression and an empty viral vector) in N2a cells and verified the overexpression efficiency of Brn4 using Western blot (Figures 2(b) and 2(c)) and RT-qPCR (Figure 2(d)). Then, the mRNA levels of the 16 overlapping genes were detected by RT-qPCR. The results showed that the transcription levels of three genes, Lama2, Pla2g4a, and Samsn1, were increased after Brn4 overexpression (Figure 2(e)). We constructed PCR primers using the promoter sequences of the three genes as a template. Among them, Pla2g4a has two transcripts with completely different promoters, so we designed different primers for the two promoters to analyze independently. ChIP assay was performed on the Brn4 overexpressing stable strain. The results indicated that Brn4 bound to the promoters of Lama2 and Samsn1, while there was no obvious Brn4 binding site in the promoters of Pla2g4a (Figure 2(f)). These results indicated that Brn4 may bind to the promoters of Lama2 and Samsn1 and promote their mRNA expression.

Next, we analyzed the binding sites of Lama2 and Samsn1 promoter regions with Brn4 using the JASPAR website. The top three sites with the highest scores were, respectively, selected as targets (Figure 2(g)), which were deleted simultaneously to construct dual luciferase reporter gene mutation plasmids. The results of the dual luciferase reporter gene experiment showed that the top three sites analyzed by the JASPAR website contained the effective Brn4-bound sites of the Lama2 and Samsn1 promoters (Figures 2(h) and 2(i)). Western blot demonstrated that, consistent with the mRNA, the level of Lama2 and Samsn1 protein also increased significantly after Brn4 overexpression (Figures 2(j)–2(m)). The above results revealed that Brn4 bound to the specific sites of the Lama2 and Samsn1 promoter regions and promoted their expression at the protein level.

### 3.3. Lama2 And Samsn1 Partially Mediated the Inhibitory Effect of Brn4 on NSC Proliferation

**Figure 3:**
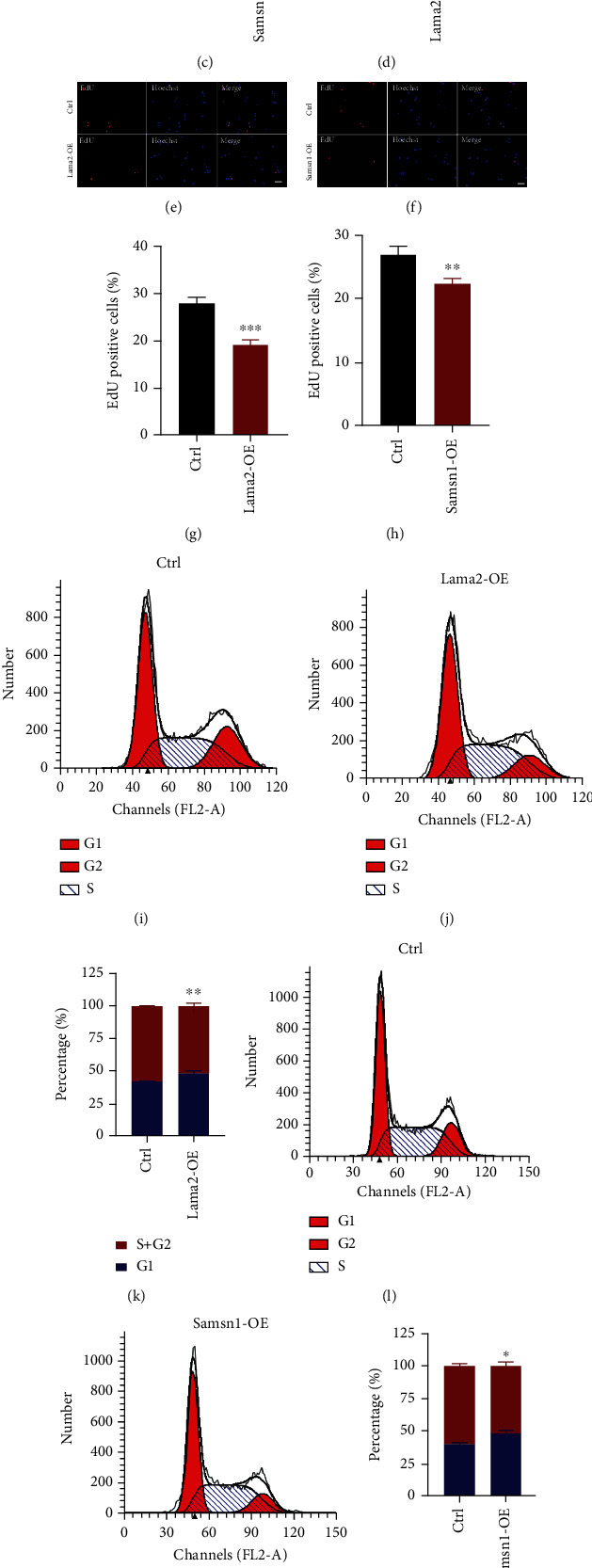
Lama2 activation or Samsn1 overexpression inhibits the proliferation of hippocampal NSCs. (a) Quantification of Lama2 mRNA level normalized to *β*-actin in the hippocampal NSCs which were transfected with different concentrations of Lama2 CRISPR SAM coactivated plasmid. (b) Quantification of Samsn1 mRNA level normalized to *β*-actin in the hippocampal NSCs which were infected by Samsn1 overexpression lentivirus. (c) The upper panel is the representative immunoblots of Samsn1 and *β*-actin after being infected by Samsn1 overexpression lentivirus; the lower panel is the quantification of Samsn1 protein level normalized to *β*-actin. (d) The upper panel is the representative immunoblots of Lama2 and *β*-actin after being infected by Lama2 CRISPR SAM coactivated plasmid; the lower panel is the quantification of Lama2 protein level normalized to *β*-actin. (e, f) Representative EdU-staining in primary hippocampal NSCs. Bar = 20 *μ*m. (g, h) Quantification of EdU-positive cells normalized to Hoechst. (i–k) Representative cell cycle distribution analyzed by flow cytometry in primary hippocampal NSCs after Lama2 activation (i, j) and quantification for NSCs in the different stages of the cell cycle (k). (l–n) Representative cell cycle distribution analyzed by flow cytometry in primary hippocampal NSCs after Samsn1 overexpression (l, m) and quantification for NSCs in the different stages of the cell cycle (n). (o, p) CCK-8 assay in primary hippocampal NSCs 0, 1, 2, 3, 4 days after plasmid transfection or virus infection. Error bars represent the mean ± SEM. *n* = 3. ^∗^*P* < 0.05, ^∗∗^*P* < 0.01, ^∗∗∗^*P* < 0.001. Abbreviations: Ctrl: control; Lama2-OE: Lama2-overexpression; Samsn1-OE: Samsn1-overexpression.

Firstly, we used Lama2 CRISPR SAM coactivated plasmids and Samsn1 overexpression lentiviruses to activate or force their expression. RT-qPCR results showed that the optimal transfection concentration of Lama2 plasmids was 1/3 *μ*g/mL medium (Figure 3(a)). Western blot showed that the level of Lama2 protein was also upregulated after activation using the Lama2 CRISPR SAM coactivated plasmids (1/3 *μ*g/mL medium, Figure 3(d)). Samsn1 overexpression lentivirus also significantly increased its mRNA and protein expression levels (Figures 3(b) and 3(c)). Compared with the control group, the proportion of EdU positive cells decreased significantly after Lama2 activation or Samsn1 overexpression in primary hippocampal NSCs, indicating that the cell proliferation rate was slowed down by Lama2 and Samsn1 forced expresssion (Figures 3(e)–3(h)). Cell cycle analysis by flow cytometry revealed that after Lama2 activation or Samsn1 overexpression, the proportion of cells in the G1 phase increased, while the proportion of cells in the S phase and G2 phase decreased, suggesting that the cell division ability was reduced (Figures 3(i)–3(n)). CCK-8 assay was performed to value the growth of the cells at 0, 1, 2, 3, and 4 days after plasmid transfection or lentivirus infection. The results showed that the value of CCK-8 declined in time-dependent manner after Lama2 activation or Samsn1 overexpression compared with the control group, supporting the above result that Lama2 or Samsn1 could inhibit the proliferation of hippocampal NSCs (Figures 3(o) and 3(p)).

**Figure 4:**
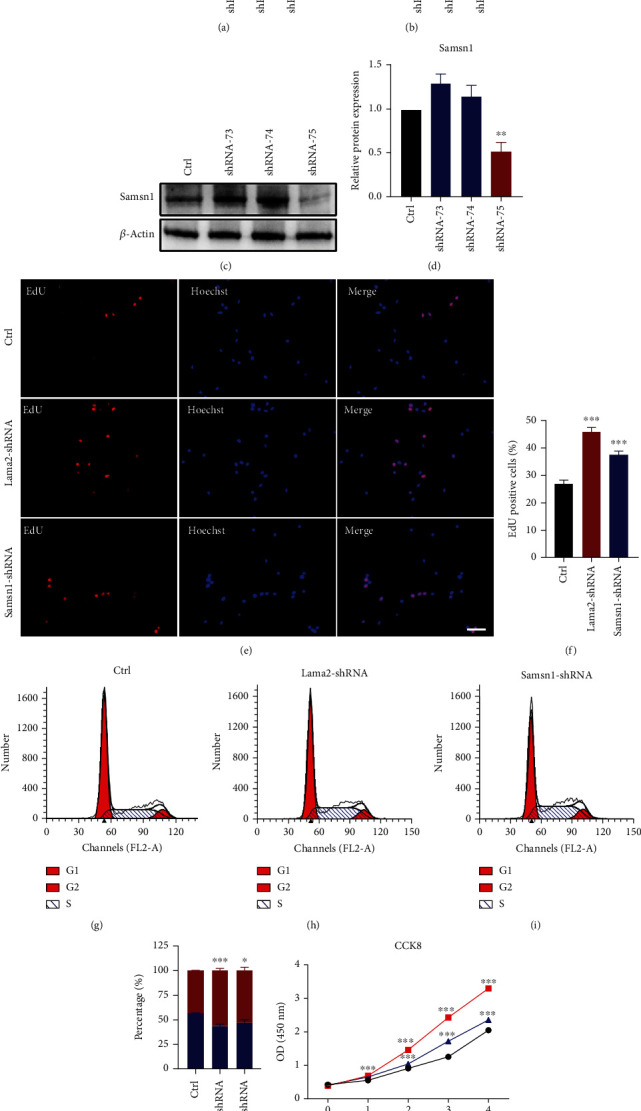
Lama2 or Samsn1 knockdown promotes the proliferation of hippocampal NSCs. (a, b) Quantification of Lama2 and Samsn1 mRNA levels normalized to *β*-actin in the primary hippocampal NSCs which were infected with Lama2-shRNA lentivirus or Samsn1-shRNA lentivirus. (c, d) Representative immunoblots of Samsn1 and *β*-actin after being infected by Samsn1-shRNA lentivirus (c) and quantification of Samsn1 protein level normalized to *β*-actin (d). (e) Representative EdU-staining after Lama2 or Samsn1 knockdown in primary hippocampal NSCs. Bar = 20 *μ*m. (f) Quantification of EdU-positive cells normalized to Hoechst. (g–j) Representative cell cycle distribution analyzed by flow cytometry after Lama2 or Samsn1 knockdown in primary hippocampal NSCs (g–i) and quantification for NSCs in the different stages of the cell cycle (j). (k) CCK-8 assay in primary hippocampal NSCs 0, 1, 2, 3, and 4 days after lentivirus infection. Error bars represent the mean ± SEM. *n* = 3. ^∗^*P* < 0.05, ^∗∗^*P* < 0.01, ^∗∗∗^*P* < 0.001.

Next, RT-qPCR and Western blot were performed to detect the knockdown efficiency of Lama2 or Samsn1 shRNA lentivirus. The results indicated that shRNA-78 and shRNA-75, one of the three shRNA sequences of Lama2 and Samsn1, respectively, had the best knockdown efficiency (Figures 4(a)–4(d)), which were used and named Lama2-shRNA group or Samsn1-shRNA group in the subsequent experiments. Compared with the control group, the number of EdU positive cells increased significantly after Lama2 or Samsn1 knockdown in primary hippocampal NSCs, suggesting that the cell proliferation rate increased (Figures 4(e) and 4(f)). Flow cytometry cell cycle analysis results revealed that after Lama2 or Samsn1 knockdown, the proportion of cells in the G1 phase decreased, while the proportion of cells in the S phase and G2 phase increased, indicating that the ability of cell division was improved (Figures 4(g)–4(j)). CCK-8 assay showed that the number of living cells increased after Lama2 or Samsn1 knockdown compared with the control group, and this difference became more obvious with time (Figure 4(k)). These results above confirmed that Lama2 and Samsn1 had a negative effect on hippocampal NSCs proliferation.

**Figure 5:**
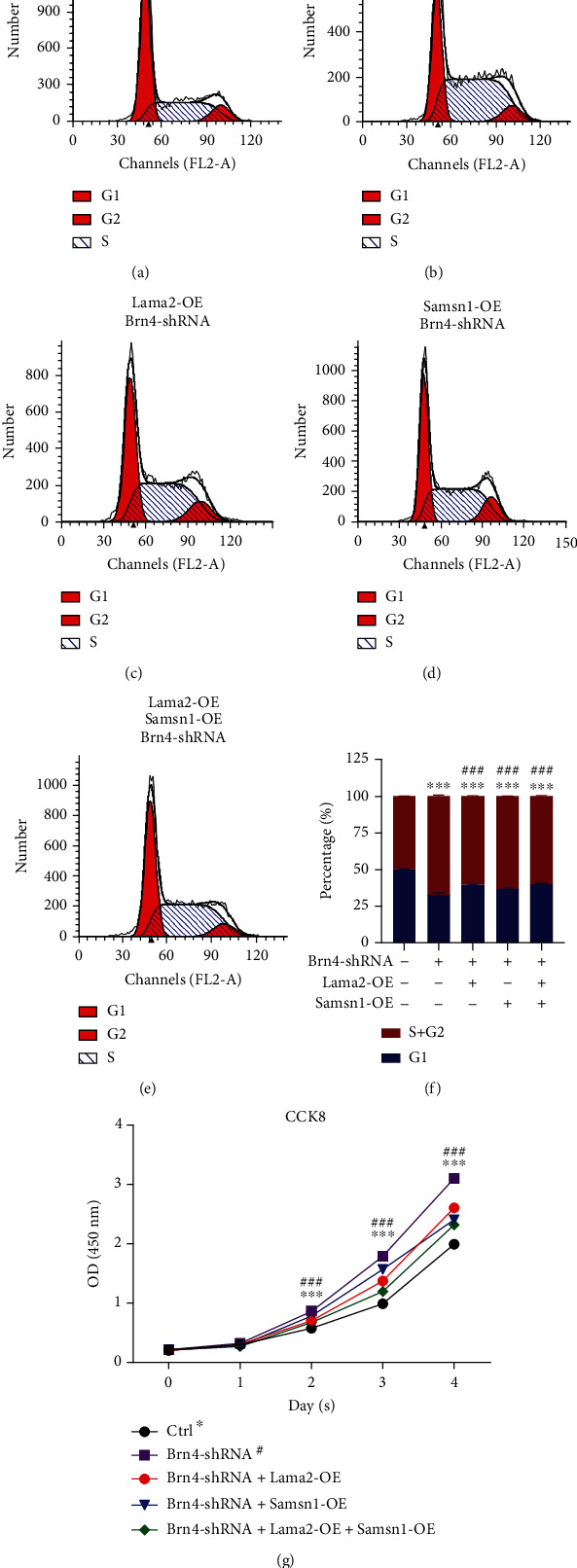
Lama2 and Samsn1 partially reverse the inhibitory effect of Brn4 on NSCs proliferation. (a–f) Representative cell cycle distribution analyzed by flow cytometry for the primary hippocampal NSCs with different treatments at the same time (a–e) and quantification for NSCs in the different stages of the cell cycle (f). (g) CCK-8 assay in primary hippocampal NSCs 0, 1, 2, 3, and 4 days after lentivirus infection or plasmid transfection simultaneously. Error bars represent the mean ± SEM. *n* = 3. ^∗∗∗^*P* < 0.001*vs.* Ctrl, ^###^*P* < 0.001*vs.* Brn4-shRNA.

Then, we investigated the roles of Lama2 and Samsn1 in the Brn4-knockdown NSCs. The results of cell cycle analysis by flow cytometry showed that after Brn4 knockdown, the proportion of cells in the G1 phase decreased, while the proportion of cells in the S phase and G2 phase increased. However, activation of Lama2 and overexpression of Samsn1 reversed the increase of cell proportion in the S phase and G2 phase induced by Brn4 knockdown (Figures 5(a)–5(f)). Similarly, Brn4 knockdown increased the CCK-8 value of NSCs which was partially reversed by Lama2 activation and Samsn1 overexpression (Figure 5(g)). All the above results confirmed that Lama2 activation and Samsn1 overexpression could inhibit the enhancement of cell proliferation induced by Brn4 knockdown, which indicated that they partially mediated the negative effect of Brn4 on hippocampal NSCs proliferation.

### 3.4. Lama2 And Samsn1 Promoting Effect of Brn4 on the Differentiation of NSCs into Neurons

**Figure 6:**
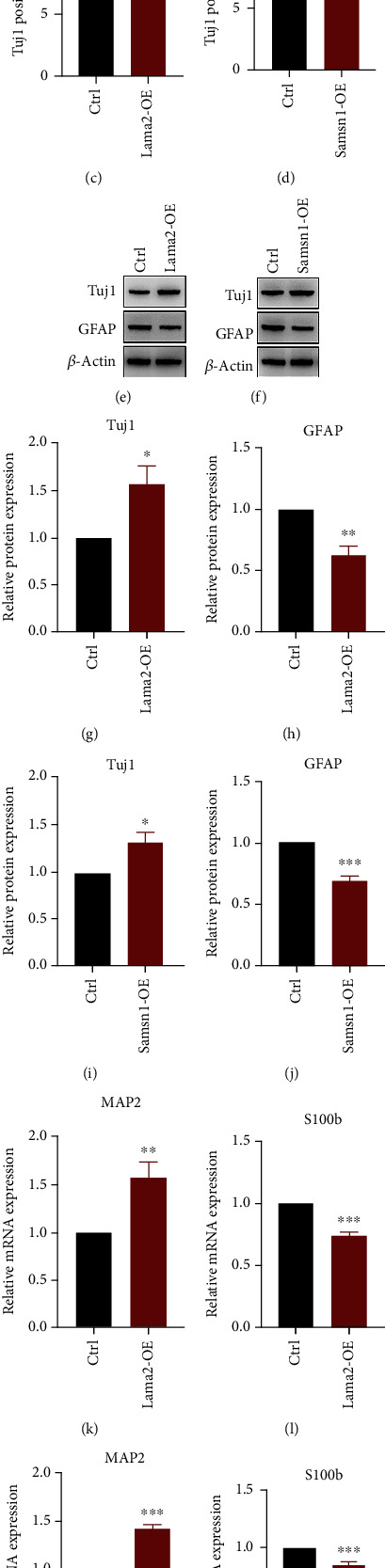
Lama2 activation or Samsn1 overexpression promotes NSCs differentiation into neurons. (a, b) Representative immunostaining for Tuj1 (red) in the primary hippocampal NSCs after induced differentiation for 7 days following plasmid transfection or lentivirus infection. Bar = 50 *μ*m. (c, d) Quantification of Tuj1-positive cells normalized to Hoechst. (e–j) Representative immunoblots of Tuj1, GFAP, and *β*-actin in the primary hippocampal NSCs after induced differentiation for 7 days following plasmid transfection or lentivirus infection (e, f) and quantification of Tuj1 and GFAP protein levels normalized to *β*-actin (g–j). (k–n) Quantification of MAP2 and S100b mRNA levels normalized to *β*-actin after induced differentiation for 7 days following plasmid transfection or lentivirus infection. Error bars represent the mean ± SEM. *n* = 3. ^∗^*P* < 0.05, ^∗∗^*P* < 0.01, ^∗∗∗^*P* < 0.001.

**Figure 7:**
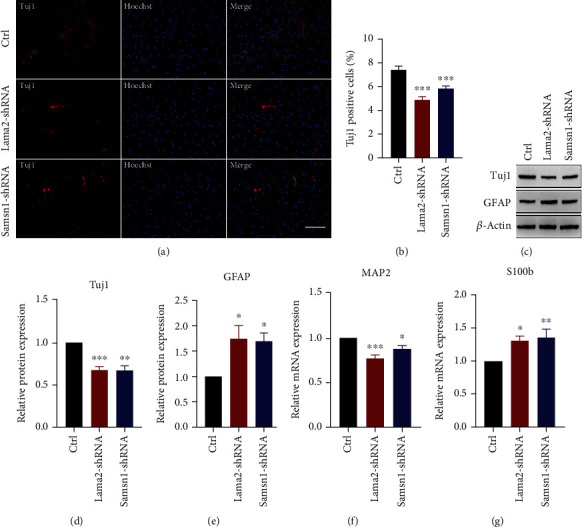
Lama2 or Samsn1 knockdown inhibits NSCs differentiation into neurons. (a) Representative immunostaining for Tuj1 (red) in the primary hippocampal NSCs after induced differentiation for 7 days following lentivirus infection. Bar = 50 *μ*m. (b) Quantification of Tuj1-positive cells normalized to Hoechst. (c–e) Representative immunoblots of Tuj1, GFAP, and *β*-actin in the primary hippocampal NSCs after induced differentiation for 7 days following lentivirus infection (c) and quantification of Tuj1 and GFAP protein levels normalized to *β*-actin (d, e). (f, g) Quantification of MAP2 and S100b mRNA levels normalized to *β*-actin after the NSCs induced differentiation for 7 days following lentivirus infection. Error bars represent the mean ± SEM. *n* = 3. ^∗^*P* < 0.05, ^∗∗^*P* < 0.01, ^∗∗∗^*P* < 0.001.

In this study, we used neuron-specific class III beta-tubulin (Tuj1) and microtubule-associated protein 2 (MAP2) as neuron markers, glial fibrillary acidic protein (GFAP), and S100 protein subunit B (S100b) as astrocyte markers. Immunofluorescence staining results showed that the proportion of Tuj1 positive cells in the Lama2 activation or Samsn1 overexpression group was higher, indicating that the number of neurons increased, than that in the control group after NSC differentiation was induced by differentiation medium composed of DMEM/F-12 and 1% FBS (Figures 6(a)–6(d)). Western blot and RT-qPCR also demonstrated that the protein and mRNA levels of Tuj1 and MAP2 elevated, while GFAP and S100b were reduced after Lama2 activation or Samsn1 overexpression (Figures 6(e)–6(n)). These results suggested that Lama2 and Samsn1 could promote the differentiation of hippocampal NSCs into neurons. Oppositely, Lama2 or Samsn1 knockdown induced the decrease in the number of Tuj1 positive cells (Figures 7(a) and 7(b)) and the protein or mRNA levels of Tuj1 and MAP2, and the increase of GFAP and S100b (Figures 7(c)–7(g)), indicating that Lama2 or Samsn1 knockdown could inhibit the differentiation of hippocampal NSCs into neurons, but enhance their ability to differentiate into astrocytes.

**Figure 8:**
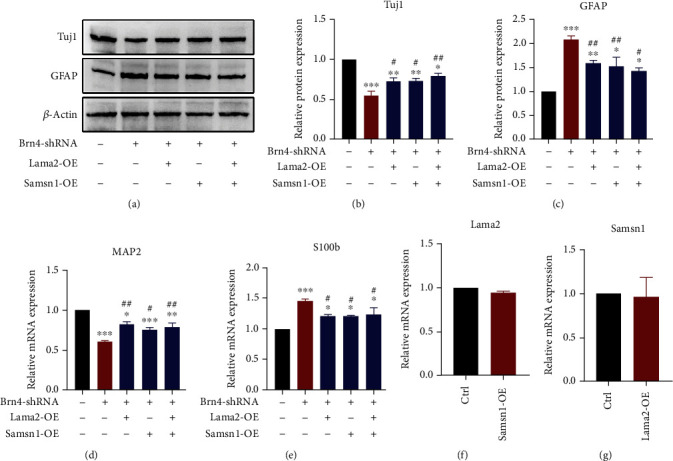
Lama2 and Samsn1 partially reversed the decrease of NSCs differentiation into neurons caused by Brn4 knockdown. (a–c) Representative immunoblots of Tuj1, GFAP, and *β*-actin in the primary hippocampal NSCs after induced differentiation for 7 days (a) and quantification of Tuj1 and GFAP protein levels normalized to *β*-actin (b, c). (d, e) Quantification of MAP2 and S100b mRNA levels normalized to *β*-actin after the NSCs induced differentiation for 7 days. (f, g) Quantification of Lama2 and Samsn1 mRNA levels normalized to *β*-actin. Error bars represent the mean ± SEM. *n* = 3. ^∗^*P* < 0.05, ^∗∗^*P* < 0.01, and ^∗∗∗^*P* < 0.001*vs.* Ctrl; ^#^*P* < 0.05 and ^##^*P* < 0.01*vs.* Brn4-shRNA.

The results of Western blot and RT-qPCR also showed that Lama2 and Samsn1 could reverse the expression levels of neuron and astrocyte markers which changed after Brn4 knockdown (Figures 8(a)–8(e)). The above results suggested that Lama2 and Samsn1 partially mediated the promoting effect of Brn4 on the differentiation of hippocampal NSCs into neurons. To explore whether there was some connection between Lama2 and Samsn1, we detected the mRNA level of Lama2 and Samsn1 after another gene activation or overexpression. RT-qPCR results suggested that Samsn1 overexpression could not change Lama2 transcription and vice versa (Figures 8(f) and 8(g)), which indicated that each of them possibly mediated the role of Brn4 in an independent way.

## 4. Discussion

Transcription factor Brn4 is widely expressed in the neural crest and neuroectoderm at the early stage of embryonic nervous system development, while in the hypothalamus, pituitary, and hippocampus at the middle stage [14]. It plays an essential role in the embryonic development of the nervous system, inner ear, and pancreas [28–30]. Our previous studies (unpublished data) have shown that Brn4 can regulate the proliferation and differentiation of hippocampal NSCs, but its downstream mechanism remains unclear.

To explore its target genes, we performed RNA-seq and ChIP-seq after Brn4 overexpression in N2a cells. N2a cells are derived from mouse neuroblastoma which have similar bioactivities as the NSCs and can also be induced to differentiate into neuron-like cells. At present, this cell line is mainly used as NSC or neural progenitor model for pathological research *in vitro* [31]. In our study, we used N2a cells as a tool cell, just to preliminarily explore the possible downstream genes of Brn4, and then verify them on hippocampal NSCs. After RNA-seq and ChIP-seq, we found 16 genes at the intersection of the two sequencing results. Next, among the 16 genes, we verified Brn4 bound to the promoters of Lama2 and Samsn1 and promoted their expression both at mRNA and protein levels using RT-qPCR, ChIP assay, dual luciferase reporter gene experiment, and Western blot. In order to investigate whether Lama2 and Samsn1 regulate the proliferation and differentiation of hippocampal NSCs, we conducted EdU incorporation, cell cycle analysis by flow cytometry, and CCK-8 assay to detect NSC proliferation ability and Tuj1 immunofluorescence staining, RT-qPCR, and Western blot to detect NSCs differentiation ability after overexpression or knockdown of Lama2 and Samsn1. The results suggested that Lama2 and Samsn1 not only inhibited hippocampal NSCs proliferation but also enhanced their differentiation into neurons. Based on the similar effects on NSCs, we proposed a hypothesis that Lama2 and Samsn1 mediated the function of Brn4 on the proliferation and differentiation of hippocampal NSCs. Thus, we forced their expression in Brn4 knockdown hippocampal NSCs, and the results showed that Lama2 and Samsn1 reversed the changes occurring after Brn4 knockdown. Interestingly, simultaneous force-expression of Lama2 and Samsn1 did not have a better effect, and Samsn1 overexpression could not change Lama2 transcription and vice versa, which indicated that they mediated the role of Brn4 in different ways.

Lama2 is the protein-coding gene of the extracellular matrix laminin *α*2 chain [32]. It not only participates in the formation of the glial vascular basement membrane of the blood-brain barrier but also exists in the neurons of the brain, regulating the synaptic function and plasticity of the central nervous system [33]. However, the role of Lama2 in the proliferation and differentiation of hippocampal NSCs has not been reported. Our results show that Lama2 have a negative effect on the proliferation of hippocampal NSCs, keeping consistency with its roles in the malignant progression of lung adenocarcinoma [34], pituitary adenoma [35], hepatocellular carcinoma [36], and colorectal cancer [37]. In addition, De La Fuente et al. [38] believe that Lama2 derived from pericytes can promote the differentiation of oligodendrocyte progenitor cells, which may explain why patients with Lama2-associated muscular dystrophy are often accompanied by myelin dysplasia. It is also proved that Lama2 plays a positive effect on the osteogenic differentiation of mesenchymal stem cells [39] and dental follicle cells [40] and the differentiation of human embryonic stem cells into islet B cells [41]. These studies more or less support our results that Lama2 promoted hippocampal NSC differentiation into neurons.

Samsn1, alias HACS1, NASH1, and SLy2, is mainly expressed in normal hematopoietic tissues and malignant tumors of the hematopoietic system, such as multiple myeloma, lymphoma, etc., and also in gallbladder, heart, lung, brain, and other tissues [42]. The expression of Samsn1 is raised after antigens stimulate B lymphocytes and then regulates the formation of cytoskeleton and membrane folds through Rac1, which in turn mitigates the diffusion and polarization of B cells to maintain the appropriate adaptive immunity [43]. Even supposing that the increased expression of Samsn1 in microglia induced by amyloid *β* may be involved in the pathogenesis of AD [44], the role of it on hippocampal NSCs remains unclear. Unlike the earlier findings from Yan et al. [45] that Samsn1 high expression is related to the poor prognosis in glioblastoma, our results find that it inhibits the hippocampal NSC proliferation, which is in good agreement with its role, as a tumor suppressor, in gastric cancer, lung cancer, and liver cancer [46–48]. Samsn1 is an adaptor protein, and its protein structure can be divided into three parts: the N-terminal is the nuclear localization signal, the C-terminal is the SAM domain, and the middle link is the SH3 domain which contributes to different proteins combined by Samsn1 acting together in the cytoplasm or nucleus [49]. Thus, this inconsistency may be due to some key molecules in glioblastoma interacting with each other to promote the malignant progression. Besides, our research indicates that Samsn1 promotes hippocampal NSC differentiation into neurons, which compares well with the previous study that Samsn1 knockdown in induced pluripotent stem cells causes the impaired differentiation ability into kidney-like cells [50].

**Figure 9:**
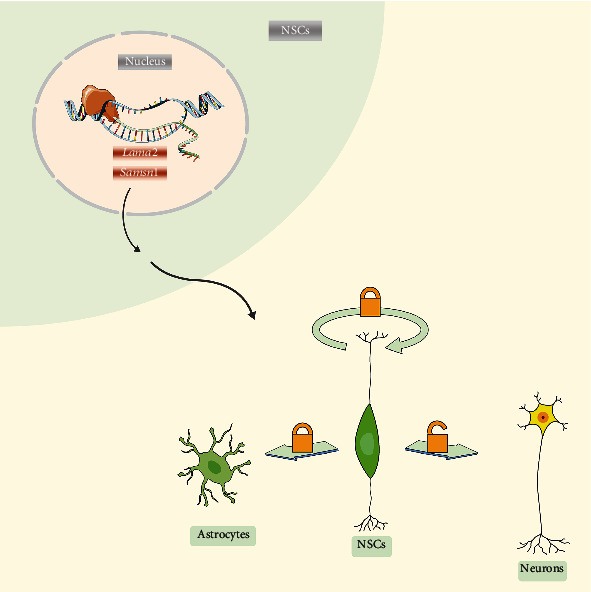
Schematic diagram describing the mechanism of Lama2 and Samsn1-mediated Brn4 effect on differentiation of hippocampal NSCs.

Our previous reports [15–18] and the result in this study indicated that Brn4 regulates the proliferation and neuronal differentiation of hippocampal NSCs. Promoting the transcription of the downstream genes Lama2 and Samsn1 may partially mediate the Brn4 effects on NSCs (as shown in Figure 9). However, the proliferation and differentiation functions of NSCs are detected mainly *in vitro*, and it is still unknown whether the results can be applied *in vivo*. The valuation of the differentiation function of NSCs is according to the number of newborn neurons and the expression level of related markers, which cannot explain whether the newborn neurons can generate and conduct excitement, form a synaptic connection with the surrounding cells after transplantation, and truly replace the damaged neurons. What is more, the downstream signaling molecules and pathways involved are still unclear, although we confirmed that Lama2 and Samsn1 mediated the regulation of Brn4 on NSCs. The above problems need to be solved urgently, and our further research direction.

## 5. Conclusion

In conclusion, our study indicated that Brn4 bound to the promoters of Lama2 and Samsn1 which may partially mediate the regulation of Brn4 on the proliferation and differentiation of mouse hippocampal NSCs. Our data provided novel insights for regulating neurogenesis of hippocampal NSCs, which is helpful to solve hippocampal disorders through self-repairing and cell replacement therapy.

## Figures and Tables

**Table 1:** The primer sequences used in the ChIP assay.

Gene symbols	Primers
Lama2	Forward: GCTTCCAACCAGTGGAGTGAT
	Reverse: GACTGTCCCAAGGTCATGGT
Pla2g4a-p1	Forward: CCCTAGGCAACTGATGTGCT
	Reverse: AAGCCCATTGCCTCTGTGTT
Pla2g4a-p2	Forward: GTGACTTGATCCGTGGCACT
	Reverse: GCAGGGTCTTATGTAGCCCA
Samsn1	Forward: CAGGCCATTTAACCAGAGCC
	Reverse: GTGATGCTACCGACTGCTCA

**Table 2:** The primer sequences used in the RT-qPCR.

Gene symbols	Primers
*β*-Actin	Forward: CTGAGAGGGAAATCGTGCGT
	Reverse: AGGAAGGCTGGAAAAGAGCC
Brn4	Forward: GACGCCAACCTCTGATGAGT
	Reverse: TACCATACAGTGTGCCCAGC
Arhgap30	Forward: TGCAGAGTTTGTGCAAGAGT
	Reverse: TGGGAGTTCTCTGAAGTAGGC
Col5a2	Forward: GAGTGCTGTCCGGTGTGC
	Reverse: ACGACCGCGTATACCTGTTA
Ddr2	Forward: ACGAAAGTGCTACCAACGGT
	Reverse: AGTAGACAGCAGTGGGTTCC
Dock2	Forward: CATGACCGGATGGAGGAGTG
	Reverse: CTTTCTGCGGGGACTTTGCT
Efemp1	Forward: CCAGATGCTTGCAAAGGTGGA
	Reverse: CAGTGGTGGCACCTGACGAA
Efhc1	Forward: GTCTGTCATCGAGCCCGTAG
	Reverse: TCGGAAGGTTTTGCCGTACA
Fyb	Forward: GGGAGTAACCCGACAGAGGA
	Reverse: CTGCCAAGGGTGGTTTTGTG
Irx1	Forward: ACGAGCGTGATGGCGAC
	Reverse: GGCGAATCTTGAGACTTGAGTG
Lama2	Forward: GAAGTGCATCTCTCCTCGGG
	Reverse: CACTTCGATGGGCTGTTCCT
Pla2g4a	Forward: GCACTGTATGAGTCGGGGATT
	Reverse: CTCCTCGGGACCTTTCTCTG
Pros1	Forward: GTCCTTGGTGGATTCTCGCT
	Reverse: GCTGATCCGAGCACAGAGAT
Rgs9	Forward: CTTCGGTGTCTCTTGGAGGAA
	Reverse: TCCACTCGCATCTTGGTTGG
Rnf213	Forward: AAGACCTGCTAGTTCACGCC
	Reverse: CGACTGGAAGACCACAGTCC
Samsn1	Forward: AGCCAGCGACTCTATGGACA
	Reverse: GGCTGTCATCATCCAGTCGG
Tmem117	Forward: TGGGCATCCGCAATGAAAGT
	Reverse: GACAAGCACCACCACGGAAG
Trpm2	Forward: CCTTCGATGAGCCAGATGCT
	Reverse: AAGGCACCTTCTCGTTAGGC
MAP2	Forward: ATCGCCAGCCTCAGAACAAA
	Reverse: GGGAGGATGGAGGAAGGTCT
S100b	Forward: CGAGAGGGTGACAAGCACAA
	Reverse: CCATCCCCATCTTCGTCCAG

## Data Availability

The figures and tables data used to support the findings of this study have been deposited in the GIN (G-Node Data Infrastructure Services) repository (https://gin.g-node.org/zhangxinhua/DSC/src/master/For%20Stem%20Cells%20International).
